# Inside out Approach to Rotator State in Hydrogen-Bonded System—Experimental and Theoretical Cross-Examination in n-Octanol

**DOI:** 10.3390/ijms23042138

**Published:** 2022-02-15

**Authors:** Michał Pocheć, Katarzyna M. Krupka, Jarosław J. Panek, Kazimierz Orzechowski, Aneta Jezierska

**Affiliations:** Faculty of Chemistry, University of Wrocław, ul. F. Joliot-Curie 14, 50-383 Wrocław, Poland; 307313@uwr.edu.pl (K.M.K.); jaroslaw.panek@chem.uni.wroc.pl (J.J.P.); kazimierz.orzechowski@chem.uni.wroc.pl (K.O.)

**Keywords:** n-octanol, melting, premelting, intermolecular hydrogen bond, solid state, IR, NDE, classical MD, CPMD, PIMD

## Abstract

The experimental and theoretical description of premelting behavior is one of the most challenging tasks in contemporary material science. In this paper, n-octanol was studied using a multi-method approach to investigate it at macroscopic and molecular levels. The experimental infrared (IR) spectra were collected in the solid state and liquid phase at temperature range from −84${}^{\circ }$C to −15 ${}^{\circ }$C to detect temperature-related indicators of pretransitional phenomena. Next, the nonlinear dielectric effect (NDE) was measured at various temperatures (from −30 ${}^{\circ }$C to −15 ${}^{\circ }$C) to provide insight into macroscopic effects of premelting. As a result, a two-step mechanism of premelting in n-octanol was established based on experimental data. It was postulated that it consists of a rotator state formation followed by the surface premelting. In order to shed light onto molecular-level processes, classical molecular dynamics (MD) was performed to investigate the time evolution of the changes in metric parameters as a function of simulation temperature. The applied protocol enabled simulations in the solid state as well as in the liquid (the collapse of the ordered crystal structure). The exact molecular motions contributing to the rotator state formation were obtained, revealing an enabling of the rotational freedom of the terminal parts of the chains. The Car–Parrinello molecular dynamics (CPMD) was applied to support and interpret experimental spectroscopic findings. The vibrational properties of the stretching of OH within the intermolecular hydrogen bond were studied using Fourier transformation of the autocorrelation function of both dipole moments and atomic velocity. Finally, path integral molecular dynamics (PIMD) was carried out to analyze the quantum effect’s influence on the bridged proton position in the hydrogen bridge. On the basis of the combined experimental and theoretical conclusions, a novel mechanism of the bridged protons dynamics has been postulated—the interlamellar hydrogen bonding pattern, resulting in an additional OH stretching band, visible in the solid-state experimental IR spectra.

## 1. Introduction

Premelting, first introduced as a concept by Faraday [1,2] in the late 19th century is a phenomenon broadly examined in contemporary science [3,4,5,6,7,8]. As a deeply non-equilibrium, dynamical process, it is very hard to investigate—the underlying molecular behavior, and other parameters are, by their very nature, gradually changing [9]. It is uniform for solid-to-liquid phase transition and consists of the creation of a thin, liquid-like film on the surface of the solid. The surface premelting has most frequently been investigated for the ice–water transition [10,11,12]. It was found to take place no more than 2–4 deg. below the melting point and it is affected by surface roughness and possible intersurface interactions [7,13]. The second type of premelting behavior is rotator state (or phase) formation [14]. It takes place in the broader temperature range then at surface premelting, and it is more intricate. It is highly dependent on the type of the studied compound, the inter- and intramolecular interactions, or even the exact composition of molecules. Studies of rotator state in alkanes shows that the number of carbon atoms in the hydrocarbon chain (odd/even) could determine its presence or absence [15]. Premelting as a whole can start as a disruption in the chains of single molecules and ends with formation of a quasi-liquid phase on the surface. So, the description needs to implement observations from all these mechanisms [13,16,17].

In the current study, we have applied two experimental methods—infrared spectroscopy (IR) and the nonlinear dielectric effect (NDE)—to shed light onto the premelting phenomenon in n-octanol.

**Figure 1:**
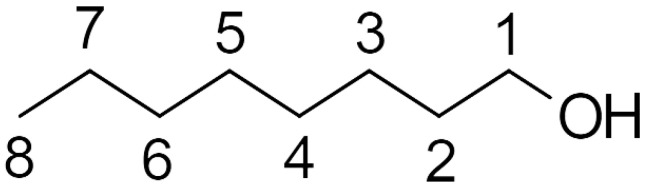
n-octanol molecule with carbon atoms numbering scheme.

The first method has already been used in many sources to assess the pretransitional behavior around melting, and it is an excellent candidate for cross-examination with the computational methods [18,19,20]. Among many applications, it could be used to identify chemical compounds or functional groups in various phases. Here, IR spectroscopy was applied for solid and liquid phase measurements of n-octanol at various temperatures. The molecular form of n-octanol is presented in Figure 1. It is a fatty alcohol with eight carbon atoms in the chain and OH group. In the solid state, n-octanol forms linear arrays of hydrogen bonds (HBs) with the chains, alternating between sites (herringbone pattern) [21]. On the basis of metric parameters, the intermolecular hydrogen bond present in n-octanol could be classified as middle strong [22,23,24]. This type of hydrogen bond is frequently encountered in aliphatic alcohols, and it is similar to hydrogen bonding in water, with the important distinction of having only one OH bond—thus, formation of such extensive HB networks, as in liquid water or ice, is less probable. Solid n-octanol is an excellent example of a system in which the structure and physico-chemical properties are governed by the interplay between hydrogen bonding and hydrophobic interactions of the aliphatic chains. Therefore, the IR spectroscopy method was used to investigate the vibrational properties of n-octanol, with special emphasis on the OH stretching.

The second experimental method, nonlinear dielectric effect (NDE), provides additional information on the premelting phenomena, especially as a macroscale processes, and has been proven by our previous findings to yield significant impact on the premelting analysis [25,26]. The main idea of this method is centered around the difference between low- and high-field dielectric permittivity, according to the following equation:

(1) $$\epsilon_{E}=\epsilon_{E\to 0}+\epsilon_{2}E^{2}+\ldots$$

where $\epsilon_{E}$ is the dielectric permittivity in high fields, $\epsilon_{E\to 0}$ is the low-field dielectric permittivity, and $\epsilon_{2}E^{2}$ is the high-field increment. The typical parameter describing the NDE changes is the Piekara factor, defined as Δϵ/*E*${}^{2}$ (equivalent to $\epsilon_{2}$), where Δϵ is the difference between dielectric permittivity in low and high fields. The nonlinear dielectric effect should be negative (for most typical dielectric liquids) [27,28] but can also be positive, also called anomalous. The second instance can be the result of system having possible states of high and low dipole moments, in equilibrium between each other [29]. It can also originate in coexistence with different phases (or quasi-phases), resulting in the Maxwell–Wagner effect [25,30], or near-critical phenomena [31,32,33].

Diverse quantum chemistry approaches have been applied to support and explain observed processes at the molecular level. We have focused on time evolution methods: (i) classical molecular dynamics (MD); (ii) Car–Parrinello molecular dynamics (CPMD) [34]; (iii) path integral molecular dynamics (PIMD) [35,36]. Classical molecular dynamics (MD) was applied to reproduce the precise molecular mechanisms leading to the observed macroscopic properties related to premelting. On the basis of the CPMD method, the intermolecular hydrogen bonds dynamics, as well as spectroscopic properties, were investigated. Finally, the PIMD technique was applied to demonstrate the quantum effect’s influence on the bridged proton position in the intermolecular hydrogen bond of n-octanol.

In this work, we aim to relate and cross-examine the macroscopic effects of premelting, observed in the experimental methods, to the dynamical molecular behavior examined via high-level computational studies. The use of non-standard experimental and theoretical approaches provided a comprehensive knowledge compendium of the premelting phenomenon in n-octanol.

## 2. Experimental and Theoretical Methods

### 2.1. Experimental Procedures: Infrared Spectroscopy (IR) and the Nonlinear Dielectric Effect (NDE)

The n-octanol of >99% purity was dried over A3 molecular sieves to eliminate any residual water. The melting temperature from both experiments was estimated at −15.0 ${}^{\circ }$C (258 K), standing in good agreement with the literature data [37]. The temperature ranges of the experiments were from −84 to −12 ${}^{\circ }$C (189 to 258 K) for IR and from −30 to −10 ${}^{\circ }$C (243 to 258 K) for NDE. All of the temperatures in computational methods are provided in Kelvins, and the experimental ones are provided both in ${}^{\circ }$C and Kelvins. This is due to the nature of raw data collected in the experiment—thermometers are mostly based in the Celsius scale.

The infrared (IR) spectra measurements were performed using the Nicolet Magma 860 FTIR (Fourier-transform-based spectra acquisition) spectrometer equipped with a custom temperature control accessory. The sample was prepared by squeezing a small amount of n-octanol between two KBr windows. The spectra were registered in the 600–3800 cm${}^{-1}$ range with the resolution of 1 cm${}^{-1}$, and the results were averaging of 12 scans. The experiment was conducted in the nitrogen atmosphere. The temperature was recorded using resistance thermometer located very close to the sample, with 0.5 deg. resolution of the readings, and an absolute error of 1 deg. The νOH region was convoluted into 3 bands using mixed Gaussian–Lorentzian distribution. Two sharp bands located on the higher wavenumber part of the approximated region were used to carry out a detailed analysis of the premelting processes. The wider bands nature is discussed later in the text.

The nonlinear dielectric effect was measured using a double-field method. The low-amplitude (1V pp), high-frequency measuring field (approx 4 MHz) was combined with a high-amplitude, quasi-rectangular polarizing field (HV), with a duration time of HV pulses at 4 ms and an amplitude of 6.25 × 10${}^{5}$ V/m. The investigated sample was poured into the parallel-plate capacitor, made of circular stainless steel electrodes of 19 mm diameter, separated by 0.35 mm. The capacitor was filled with liquid alcohol at room temperature and placed in the dry air chamber, which was submerged in a liquid bath thermostat. In the course of the NDE experiment, the temperature was measured by a calibrated resistance thermometer located close to the sample. Resolution of temperature readings was of 0.01 deg., with an absolute error of 0.2 deg. At the beginning of the experiment, the sample was thermostated for at least 3 h at −40 ${}^{\circ }$C and then the temperature was slowly increased by 0.2 deg/min. Details of NDE equipment are presented elsewhere [38].

### 2.2. Computational Methodology

#### 2.2.1. Classical Molecular Dynamics (MD)

Molecular dynamics (MD) with classical force fields was used to study the conformational behavior of the n-octanol molecules in the crystalline phase. The numbering of carbon atoms within a molecule, which follows the systematic chemical notation, is depicted in Figure 1. This numbering is used throughout this study, especially in the context of dihedral angles.

**Figure 2:**
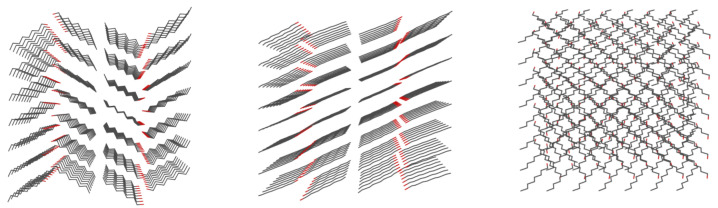
n-octanol molecules cluster used in classical dynamics experiment, as visible along different axes—on-plane (**left**), in-plane (**center**), and cross-plane axes (**right**).

The models for classical MD studies were prepared on the basis of crystal structure deposit CCDC code 263655 [21]. For the classical molecular dynamics, 8 × 7 × 1 supercells, containing 224 molecules, were prepared on the basis of crystalline structure using the Mercury 2020.1 program [39], so that the supercell dimensions were as close to cubic as feasible within the assumed system size (initial values, further subject to change by the use of barostat scheme: 33.65 × 36.29 × 38.94 Å). An exact spatial composition for this system is depicted in Figure 2.

Further setup for the classical MD simulations was as follows: the general organic GAFF force field [40] was used, and the real space non-bonded interaction cutoff was set to 10 Å. The reciprocal space summation was performed with the particle mesh Ewald (PME) scheme [41,42]. An initial energy minimization with steepest descent method was carried out for 1000 steps. The usual equilibration phase was not included, because our idea was a reproduction of crystalline phase behavior under thermal impulse. Therefore, the minimization was followed by production dynamics runs in NPT ensemble for 50 ns. The variable pressure approach is meant to allow the crystalline system to melt, which happened only at the highest considered temperature. The time step of 1 fs was used for equations of motion, and temperature was controlled via the periodic rescaling scheme of Andersen [43]. The isotropic scaling Berendsen barostat [44] with target value of 1 atm was used. The simulation of the heat impulse was studied by running the NPT computations in the temperatures spanning from 237 K to 417 K with 10 K increment—in total, 22 runs. The preparation of the topology and parameter files, as well as the MD runs, were carried out using programs from the AmberTools2021 and the Amber20 suite [45]. The VMD 1.9.3 visualization system [46] was applied for post-processing of the classical MD trajectories.

#### 2.2.2. Car–Parrinello Molecular Dynamics (CPMD) in the Crystalline Phase

Car–Parrinello molecular dynamics (CPMD) [34] were carried out in the crystalline phase for n-octanol. The models were constructed on the basis of X-ray experimental data [21] with unit cell dimensions a = 4.206 Å, b = 5.184 Å and c = 38.937 Å with α = 90${}^{\circ }$, β = 91.72${}^{\circ }$, and γ = 90${}^{\circ }$. The calculations were carried out at various temperatures: 150 K, 190 K, 230 K, 257 K, 270 K, 300 K, and 350 K. The geometry optimization was performed with the Γ-point approximation (i.e., using only Bloch eigenfunctions with zero reciprocal vector k to represent the periodic states in the crystal) [47]. Furthermore, the computations were performed with periodic boundary conditions (PBC) and with real-space electrostatic summations for the eight nearest neighbors in each direction (TESR = 8). The energy minimization was performed using the Hessian matrix of Schlegel [48] with the Perdew, Burke, and Ernzerhof (PBE) functional [49] and the norm-conserving Troullier–Martins pseudopotentials [50]. The plane–wave kinetic energy cutoff was set to 100 Ry. In the next step, the Car–Parrinello molecular dynamics (CPMD) [34] simulations were performed. The time step was set to 3 a.u. and the fictitious electron mass parameter was equal to 400 a.u. The temperature was controlled by the Nosé–Hoover thermostat [51,52]. The empirical van der Waals corrections by Grimme [53] were added to reproduce the intermolecular weak forces, and the dispersion corrections were considered within the unit cell and its nearest neighbors in each direction (3 × 3 × 3 van der Waals summation). The initial parts of the CPMD runs were taken as an equilibration (30,000 steps for the solid state) and were not considered during the data analysis. The trajectories with the data used in post-processing were collected for ca. 70 ps. On the basis of the CPMD results the metric (dihedral angles and distances), as well as spectroscopic parameters, were discussed. The Fourier transformation of the autocorrelation function of dipole moments and atomic velocity was applied for the theoretical infrared spectra. The power spectra of atomic velocity were decomposed and the OH stretching was plotted (and analyzed separately to give deeper insight into spectroscopic signatures related to the presence of the intermolecular hydrogen bonds). The CPMD simulations were carried out using the CPMD ver. 4.3–4610 program [54].

#### 2.2.3. Path Integral Molecular Dynamics in the Crystalline Phase

Path integral molecular dynamics (PIMD) [35,36] simulations were performed in the crystalline phase for n-octanol molecular crystal [21]. For the computations, a similar setup to the one applied for the CPMD was used. The experimental unit cell with a = 4.206 Å, b = 5.184 Å and c = 38.937 Å with α = 90${}^{\circ }$, β = 91.72${}^{\circ }$, and γ = 90${}^{\circ }$ [21] was used during the PIMD simulations. The calculations were carried out at 190 K. For imaginary time path integration, 8 Trotter replicas (P = 8) were applied. The data were collected for 70 ps after the initial equilibration of 30,000 steps. The obtained results served to indicate the hydrogen position in the intermolecular hydrogen bridge, in particular, through preparation of a potential of mean force (pmf) profile. The PIMD simulations were performed as well using the CPMD ver. 4.3–4610 program [54].

#### 2.2.4. Post-Processing of the Car–Parrinello and Path Integral MD

The post-processing of the obtained results was carried out with the following approaches: the intermolecular hydrogen bonds dynamics was analyzed using scripts available in the VMD 1.9.3 suite of programs [46]. The Fourier transform power spectra of atomic velocity were computed using homemade scripts. The predicted spectra were computed using a script developed by H. Forbert and obtained from the CPMD website [55]. The potential of mean force (pmf) profiles for both CPMD and PIMD were calculated using homemade programs on the basis of the OH bond length distributions. Direct logarithmic approach, i.e., pmf(r) = −kT ln(ρ(r)), was assumed (where ρ(r) is the probability density for the OH distance). The VMD 1.9.3. [46], Gnuplot [56], and Mercury [39] programs were used for visualization, models preparation, and graphical presentation of the obtained data.

## 3. Results and Discussion

In the following paragraph, the experimental (IR and NDE) and computational (based on various MD schemes) results for n-octanol are analyzed and discussed. As the last part of the paragraph, a cross-examination of the obtained data is presented.

### 3.1. Experimental Data Analysis

**Figure 3:**
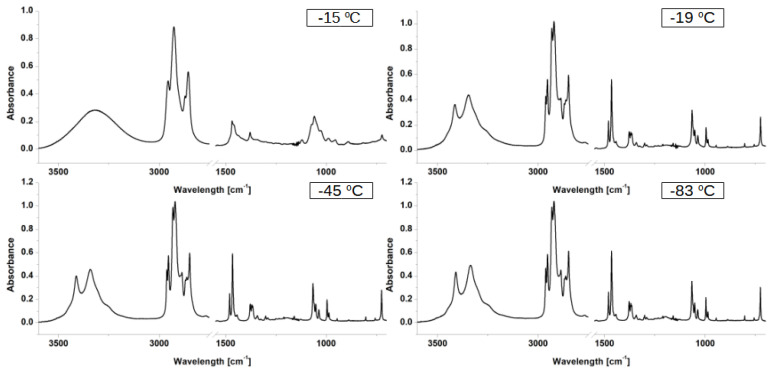
IR spectra of n-octanol measured at various temperatures. The range from 2750 to 1650 cm${}^{-1}$ was omitted for clarity due to the lack of significant bands. Liquid state spectrum: −15 ${}^{\circ }$C (258 K), solid state spectra: −19 ${}^{\circ }$C (254 K), −45 ${}^{\circ }$C (228 K), and −83 ${}^{\circ }$C (190 K).

Fifteen infrared spectra were registered in the temperature range from −83 ${}^{\circ }$C (190 K) to −15 ${}^{\circ }$C (258 K). The lowest temperature was chosen to match the temperature of the crystal data of n-octanol, deposited in the Cambridge Crystallographic Data Centre (CCDC) [57]. In Figure 3, the IR spectra measured at various temperatures are presented. The range from 2750 cm${}^{-1}$ to 1650 cm${}^{-1}$ was omitted due to the lack of significant bands. The complete set of spectra is collected in the Supplementary Materials (Figures S1–S4).

**Figure 4:**
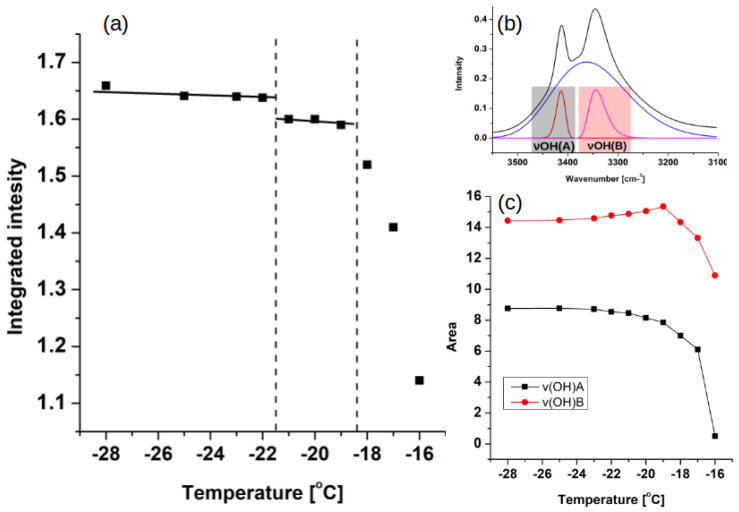
(**a**) Temperature evolution of integrated intensity of the CH${}_{2}$ rocking vibrations region (710–750 cm${}^{-1}$), (**b**) deconvolution of stretching OH (νOH) region and (**c**) temperature dependence of the area of deconvoluted νOH bands.

Predictably, the most notable differences occurred between the liquid-state spectrum and its solid-state counterparts. Herein, we will discuss the regions important for further experimental and theoretical analyses. The νOH region located between 3500 cm${}^{-1}$ and 3100 cm${}^{-1}$ in the liquid phase consists of one, broad band, while in the solid state, it is visibly split into two sharp peaks and an underlying, broader, slightly asymmetrical band. This discrepancy between number of bands between solid and liquid samples can be the result of freezing of rotational freedom in the solid state. It is also visible in other parts of the spectrum. In the solid state spectra, the CH${}_{2}$ rocking vibrations region, located between 750 cm${}^{-1}$ and 710 cm${}^{-1}$, consists of two distinct bands, while in liquid phase, only one broad band is visible. In order to assess the exact extent, magnitude and type of premelting behavior, a detailed analysis of temperature evolution for both CH${}_{2}$ rocking vibrations region and νOH region was necessary. The results of the analysis are presented in Figure 4 and the methodology of the spectra deconvolution is explained in Section 2.

The CH${}_{2}$ rocking vibrations region is an established marker sensitive to the rotator state formation [13,16]. The temperature evolution of its integrated intensity shows a double change—the first change occurs around 6 deg. before the melting, and it is visible as a shelf-like dip. It is followed by the more pronounced drop around 2 deg. before melting. The first change can be attributed to the onset of rotator state formation, while the second one is consistent with the surface premelting process. The auxiliary analysis of the changes in the νOH region follows the same pattern—the two-fold change pronounces the observations from the CH${}_{2}$ rocking vibrations region analysis.

**Figure 5:**
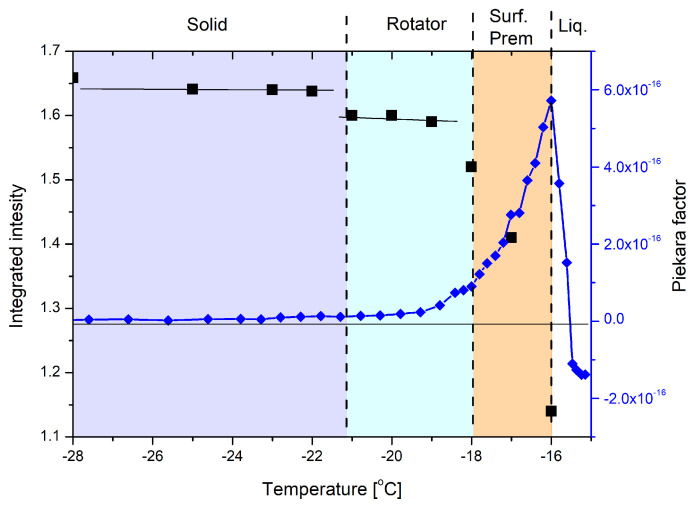
Comparison between temperature evolution of integrated intensity of the CH${}_{2}$ rocking vibrations region (black) and the NDE results, given as Piekara factor (blue).

The nonlinear dielectric effect (NDE), as mentioned before, is a method sensitive to any phase inhomogeneity. Thus, surface premelting should be visible in NDE measurements due to the Maxwell–Wagner effect [30]. The results of the NDE experiment and the comparison with data from the CH${}_{2}$ rocking vibrations region analysis are presented in Figure 5.

In the NDE experiment, the first change (in the heating run) is visible as a small increase in the Piekara factor around −23 ${}^{\circ }$C (250 K). The temperature range where the change starts to be visible corresponds with the formation of rotator state, suggested in the IR experiments. The second change starts just below −18 ${}^{\circ }$C (255 K) and is much bigger in magnitude, as expected for the surface premelting [25]. A large increase in the Piekara factor in close vicinity of the melting point was already observed [25,26] and was explained as a result of the Maxwell–Wagner effect, related to the formation of a liquid layer on the surface of solids [30]. The positive NDE effect, observed in larger distance from the melting point, needs more explanation. Positive effect means that the electric permittivity, measured at high electric field strength, is larger than that at low electric field strength. In our opinion, the effect is correlated with the appearance of the rotator phase. However, according to the IR measurements in the first step of premelting, only some hydrocarbon fragments of octanol gain a certain freedom of movement. This movement should not result in a positive NDE effect. We propose to consider a possible effect of ions being a result of auto-dissociation of alcohol or because of impurities. In ideal crystalline state migration of ions under the influence of a strong electric field is very difficult. Appearance of the rotator state and freeing of rotational freedom in parts of the chains should allow the ions to move. The movement could result in a formation of polarized domains, which, consequently, should be visible as a positive NDE effect. It seems that both methods yielded similar results and lead us to the conclusion that n-octanol exhibits two types of premelting behavior—rotator state formation and surface premelting.

### 3.2. Classical Molecular Dynamics (MD) Results

The root mean square deviation (RMSD) parameter, the simplest yet very important estimator of structural stability, was calculated for a series of classical MD trajectories in diverse temperatures. The results, shown in Figure S5 of the Supplementary Materials, show that all the simulations (with exception of the highest temperature, 417 K) are stable within the 50 ns time range. The RMSD oscillates around 1 Å with respect to the initial structure, corresponding to the experimental X-ray data [21]. Only in 1 case (367 K) is the equilibrium structure ca. 1.6 Å from the reference. The exception is the 417 K simulation, where the RMSD grows very quickly to hundreds of Å, indicating the transition to the liquid phase. This will be elaborated further in the description of the radial distribution functions (RDFs). The large difference between the experimental melting point (257 K) [37] and the conditions at which the simulation yields liquid phase is explained by the relatively short time scale of the classical MD (50 ns), in which the system must leave the free energy minima and change its density, which results in the collapse of ordering. In our case, we believe that the additional kinetic energy of the heat impulse was necessary to overcome the entropic factor, i.e., enough molecules were agitated to ensure the structural ordering collapse.

**Figure 6:**
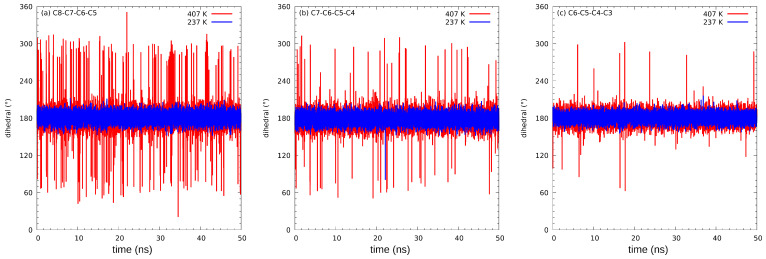
Time evolution of three dihedral angles relevant to the aliphatic chain conformation in selected molecule: (**a**) C8-C7-C6-C5, (**b**) C7-C6-C5-C4, and (**c**) C6-C5-C4-C3 angle. Results of classical MD at 237 K (blue line) and 407 K (red line).

The dihedral angles C8-C7-C6-C5, C7-C6-C5-C4, and C6-C5-C4-C3 (for details see Figure 1) indicate, in the best way, whether the chains are subjected to any conformational perturbations. We use the dihedral angle range from 0 to 360${}^{\circ }$ instead of the customary −180–+180${}^{\circ }$ range throughout the study, so that the discontinuities of the studied parameters are avoided. In Figure 6, are shown the time evolutions of dihedral angles in different temperatures for a selected molecule within the simulated ensemble; while the low-temperature simulation shows virtually no changes outside the all-trans conformational minimum, the high-temperature simulation exhibits frequent, but short-lived changes to other conformational minima. It is also evident that the outermost dihedral angle is also the most active, which might be related both to the steric effect of the presence of neighbors preventing large conformational changes, and to the larger mass of groups which are being moved when the C6-C5-C4-C3 dihedral angle is changed.

Table 1 denotes the trajectory count of rotated angles. It is clearly visible that while in the 237 K–327 K range the dihedral angles are within the baseline boundaries corresponding to the all-trans conformation, starting with the 337 K, the apparent instances in dihedral rotations occur. It is also evident that the probability of those rotations rises with the temperature. As already indicated above, the comparison between C6-C5-C4-C3, C7-C6-C5-C4, and C8-C7-C6-C5 reveals that more of the perturbations happen closer to the terminal parts of the chains—even just before melting, the rotations are almost 10 times more likely to occur within C8-C7-C6-C5 than C6-C5-C4-C3. It is also worth noting that after melting (417 K), the rotations are almost uniformly spread along the chains, with no more than 10% difference in counts. This means that the rotational freedom in the rotator state acts differently than in liquid phase. Comparing terminal (C8-C7-C6-C5) perturbations between just before and just after the melting (407 K and 417 K, respectively) reveals that the magnitude of rotator state perturbations is around 10%, which is similar to the literature data [15].

**Figure 7:**
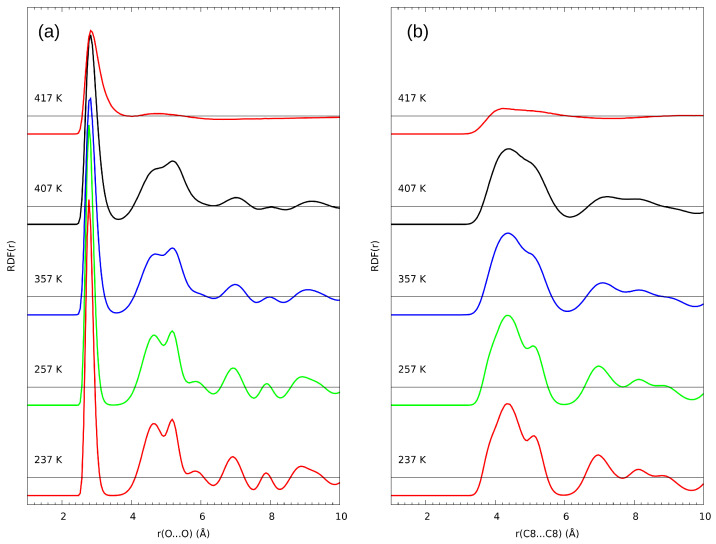
Temperature dependence of radial distribution functions (RDF) for (**a**) O–O and (**b**) C8–C8 atom pairs. Results of classical MD simulations of n-octanol in five selected temperatures.

The intermolecular distances distribution along the whole trajectory has also been analyzed as the radial distribution function (RDF), portrayed in Figure 7. It is worth noting that each peak in RDF is synonymous with different order of distances—first there will be closest in-line neighbors within a single plane, then interlamellar closest neighbors, then the one-removed, in-line neighbors, in-plane but cross-line neighbors, etc. This is especially complicated in C8–C8 distances, where in a single line of hydrogen bonds, the chains are on alternating sides of this line (Figure 2).

From this analysis, it is clearly visible that the intermolecular distances are not altered in any major way. Both the O–O and C8–C8 distances distributions are broadening, but this effect can be attributed to the rotational freedom of the terminal parts of the chains, already mentioned before. If the molecules were to lose their ordering, those parameters would be greatly altered, especially in the C8–C8 distances—which is happening at 417 K. The collapse of the solid structure is visible as the disappearance of the clearly defined, sharp peaks in the C8–C8 distances, as well as the second and further orders in the O–O distances. This behavior means that the molecules remain well ordered all the way until melting, with only rotational freedom of the chains emerging while approaching T${}_{m}$. Beyond melting, the only ordering is due to the association formed by the hydroxyl groups and visible as the first order, single peak with maximum at 2.8 Å (slightly larger than in the crystal), and a barely visible, very flat and broad secondary feature between 4.2 and 5.4 Å. The RDF profile at 417 K is in very good agreement with an earlier study on neat and hydrated liquid n-octanol [58].

### 3.3. Car–Parrinello Molecular Dynamics (CPMD)

Car–Parrinello molecular dynamics was used to study the metric and spectroscopic features of n-octanol in diverse temperatures, with emphasis on the hydrogen-bonded hydroxyl groups. It should be noted, however, that the samples did not melt in any of the applied temperatures. This is mostly due to the far shorter (when compared with classical simulations) time spans of the runs, measured in picoseconds—not nanoseconds. The conformational changes from the all-trans native conformation of the crystal were also not recorded in the CPMD runs. On the other hand, Table 2 indicates growing amplitudes of torsional motions, represented as standard deviations from the average values.

**Figure 8:**
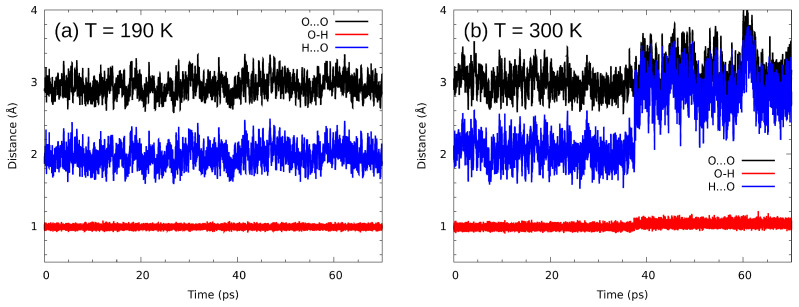
Time evolution of metric parameters related to a selected hydrogen bridge. Results of CPMD simulations in two temperatures: (**a**) 190 K and (**b**) 300 K.

CPMD, using first principles (DFT) approach to provide potential function for nuclear motions, allows for more realistic description of hydrogen bonding details, as compared with the classical force fields. We have selected a hydrogen bridge formed by one of the four molecules of the unit cell, and collected time evolution data of its metric parameters—see Figure 8. A striking feature is seen in panel (b) of this Figure. Not only the motion amplitudes are larger at an elevated temperature, but also a reorientation event happens at 38 ps of simulation time. The O-H...O contact is temporarily broken, but the O–O distance still stays within definite bounds—the OH groups do not gain complete freedom. The visible increase in the OH bond length after the reorientation event shows that the proton is now engaged into another hydrogen bridge, which is explained further in the discussion of vibrational signatures of the hydroxyl protons.

**Figure 9:**
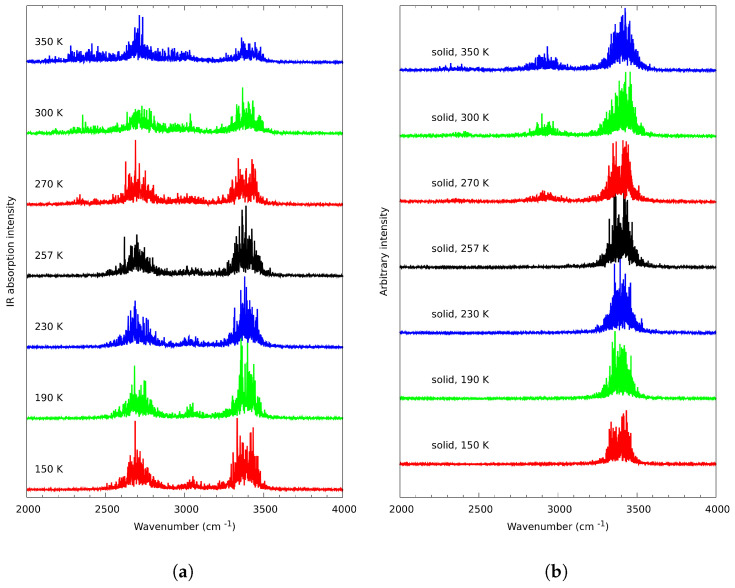
Temperature dependence of (**a**) dipole moment power spectra corresponding to the IR absorption, (**b**) vibrational signatures of the –OH proton atoms—results of Car–Parrinello molecular dynamics simulations.

The method allows acquisition of vibrational spectra, feasible in cross-examination with the IR experiment. Figure 9a,b present the spectral results of the CPMD simulations (included also in SI as Figures S6 and S7, for better clarity). Figure 9a depicts the 2000–4000 cm${}^{-1}$ region of the dipole moment power spectrum (with correct absorption intensities), while Figure 9b corresponds only to the hydroxyl proton contribution in this region, with intensities related to the motion amplitudes, not to the observable IR parameters.

In temperatures 150 K–257 K, the OH stretching band (Figure 9b) consists of a split, wide band at around 3400 cm${}^{-1}$. This band is the result of the bridged protons motions, including the cooperative effect. Above those temperatures, a third wide band around 3100 cm${}^{-1}$ arises. This band is the result of the bridged proton rotation. Considering the nature of the OH stretching vibrations, it is evident that the proton is not completely free—“free OH” stretching vibrations band would be visible above the “H-bonded” stretching vibrations band (at higher wavenumbers). The band emerging as a result of the proton rotation is located below the original band (at lower wavenumbers)—this means that the proton is still a part of some other hydrogen bridge. Considering all of the facts mentioned above, we postulate the emergence of hydrogen bonding between two different planes, which are referred to as “interlamellar OH band” in the text. The split OH stretching band is visibly shrinking in intensity (Figure 9a).

### 3.4. Path Integral Molecular Dynamics (PIMD)

**Figure 10:**
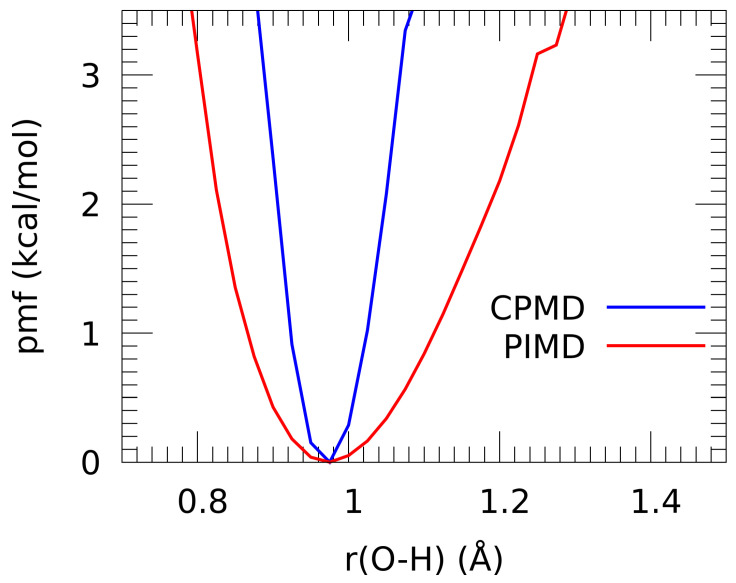
Potential of mean force (kcal/mol) for the proton motion in the hydrogen bridge. Results of CPMD and PIMD simulations—CPMD corresponds to the classical nuclear dynamics, while PIMD incorporates nuclear quantum effects.

The main purpose of the PIMD study was estimation of the role of quantum effects on the bridge proton behavior. Incorporation of nuclear quantum effects with the PIMD approach was studied by calculating the potential of mean force (pmf) for the proton motion in the intermolecular O-H...O bridge; the pmf profile is equivalent to the free energy landscape, which includes statistical sampling (entropy) effects. The comparison of classical (CPMD) and quantum (PIMD) nuclear dynamics regimes at 190 K is presented in Figure 10. It should be stressed that the terms “classical” and “quantum” refer to the nuclear dynamics; both CPMD and PIMD are based on quantum (DFT) description of the molecular electronic structure.

The most important finding is that at the temperature of 190 K the role of nuclear quantum effects is quantitative, not qualitative. The broadening of the profile with simultaneous general preservation of its shape indicates that the tunneling phenomena do not play any significant role. On the other hand, penetration of the proton wavepackets into the classically inaccessible region is quite strong and asymmetrical: the hard core region of r(OH) < 0.9 Å is significantly less explored by the proton than the region towards the acceptor atom. On the other hand, the classical nuclear approach of Car–Parrinello MD yields a very harmonic, symmetric potential. The difference between the results of CPMD and PIMD is surprisingly alleviated by the fact that for both methods the minimum is predicted at the same OH bond distance of 0.98 Å. This part can be concluded by stating that nuclear quantum effects, even at such low temperature as 190 K, are not decisive in the case of n-octanol.

### 3.5. Cross-Examination of Results

Due to the aforementioned computational limitations, the temperatures in the next section will be marked by (EX) for experimental results, (MD) for molecular dynamics results, and (CP) for CPMD, as they can not be subjected to the one-to-one comparison.

We would like to start the cross-examination with the comparison of experimental data and classical molecular dynamics results. The main goal of the latter method was confirmation and description of possible alterations in rotational and conformational freedom in chains. It is evident from the description of the classical MD results that in the 237(MD) K temperature the rotational freedom of the chains is negligible, which is expected in the solid state—the stabilizing effect of the chain–chain interactions is the dominating factor in this system. At the temperature of 337(MD) K, the perturbations in the chains become evident, as implied by an order of magnitude increase in the perturbed dihedral angles count. This behavior is even more pronounced in the 407(MD) K temperature, where the count of the perturbed angles is even bigger. At the same time, as seen in Figure 7, the O–O and C8–C8 distances are not elongated between 257(MD) K and 407(MD) K in any major way, meaning the intermolecular distances are not stretched. This indicates that the solid structure, although perturbed on the single-molecule level, is still ordered in bulk, with no increase to intra- or interlamellar distances. It is also evident that most of the instances of rotations occur in the farther (closer to terminal) parts of the chains, as seen in the C6-C5-C4-C3, C7-C6-C5-C4, and C8-C7-C6-C5 dihedral angles comparison. The collapse of the solid structure results in the sharp changes of every studied parameter. The RDF analysis reveals that the C8...C8 distances become disordered, but the first coordination sphere of the hydroxyl O atom remains similar—this is due to the association of alcohol molecules in the liquid, but random distribution of said associates occurs. The chains acquire full rotational freedom, almost uniformly spread along the whole chain. This corresponds well with the experimental results. In the analysis of the CH${}_{2}$ rocking vibrations region, the initial, shelf-like change is connected to the conformational freedom of the terminal parts of the chains. This occurs around 250(EX) K. The slight increase in the Piekara factor in NDE suggests similar reasoning—the rotations in chains could free the minuscule amounts of ionic impurities in the crystal structure, which in turn can contribute to additional, minor polarization in the electric field resulting in small, positive Piekara factor. It has been postulated in the literature that the molecules in the rotator state do not break the ordering, but some chains obtain rotational freedom along the long axis [13,16,59]. All of those factors are clear indicators that the MD description of the changes in chains is not only consistent with the experimental data, but also to the proposed molecular behavior of the rotator state.

The hydrogen-bonded hydroxyl groups are best described with the cross-examination of infrared spectroscopy and CPMD results. In both methods, the unusual shape of the stretching OH vibrations region is evident. We have suggested that in the experimental spectra it is split into three bands—two sharper and one broader (Figure 4). This pattern is also repeated in CPMD, but the bands do not overlap as much as in the experiment (Figure 9b). This is most probably the result of the so called “drag effect”, inherent to the CPMD propagation of degrees of freedom, and we believe it is not affecting each of the bands in the same way (the νCH bands are 200 cm${}^{-1}$ lower than in the experiment, while νOH are almost in exactly the same place). However, the presence of all three bands in both methods allows the assignment within the experimental spectral region to the dynamical behavior of protons, while the two sharper bands are the result of the regular hydrogen bonding between molecules (and the cooperative nature of the hydrogen bonding network), the additional band, located in the lower wavenumbers, is the result of the OH bonding pattern altering—the rotation of the hydrogen bond from one of the lines. To our knowledge, this is the first time such interlamellar hydrogen bonding has been postulated. Additionally, the changes in intensities of the two sharper bands (Figure 5 and Figure 9a) are similar between experiments. Both methods showed that the intensities of those bands are diminishing while the premelting is progressing.

It is also worth noting that the OH groups remain ordered. This is confirmed by the CPMD simulations (Figure 9a)—the system remains ordered, even though rotator state formation results in changes in the bridged protons dynamics.

## 4. Conclusions

n-octanol was the subject of combined experimental and theoretical investigation with the ultimate aim of shedding light on the premelting phenomena. The experimental studies in diverse temperatures have revealed that the system undergoes a two-step pretransitional change. The first one, starting around 10 deg. below the melting point, has been interpreted as rotator state formation. The second one, spanning 3–4 deg. below melting, has been identified as surface premelting. The exact molecular behavior of rotator state in n-octanol has been studied using classical molecular dynamics. Significant increase in the rotational freedom in the terminal parts of the chains was recorded. The RMSD and RDF have been used as indicators of crystalline ordering in the rotator state. The Car–Parrinello MD has shown the dynamical reasons of the composition of the OH stretching vibrations region. The interlamellar hydrogen bond has been postulated as a factor behind the presence of the additional, unusual band. Inclusion of nuclear quantum effects using path integral MD has revealed that the proton position in the hydrogen bridge becomes more delocalized, without significant qualitative changes to the proton potential profile.

## Figures and Tables

**Table 1:** Number of instances of the indicated dihedral angles being outside the 120${}^{\circ }$–240${}^{\circ }$ range that the region of the native all-trans conformer. Three selected molecules were chosen, and the maximum count is 30,000 frames. Results of classical MD simulations.

Temp. (K)	Dihedral Angle		
	C8-C7-C6-C5	C7-C6-C5-C4	C6-C5-C4-C3
237	3	1	0
247	3	0	0
257	4	1	0
267	13	1	0
277	12	1	0
287	17	3	0
297	21	8	0
307	49	6	0
317	81	12	4
327	65	13	1
337	122	23	5
347	139	38	2
357	206	58	3
367	360	97	20
377	655	641	142
387	459	175	43
397	626	194	79
407	846	311	93
417	11,799	10,093	9972

**Table 2:** Temperature dependence of the indicated dihedral angles: arithmetic mean ± standard deviation along the trajectory. Results of Car–Parrinello MD simulations.

Temp. (K)	Dihedral Angle (${}^{\circ }$)		
	C8-C7-C6-C5	C7-C6-C5-C4	C6-C5-C4-C3
150	178.77 ± 4.29	180.70 ± 3.48	180.05 ± 3.70
190	178.62 ± 4.80	180.85 ± 3.85	179.79 ± 4.22
230	179.00 ± 5.35	180.69 ± 4.16	179.98 ± 4.54
257	178.79 ± 5.69	180.87 ± 4.46	179.79 ± 4.90
270	178.51 ± 5.74	180.92 ± 4.58	179.93 ± 5.04
300	178.92 ± 6.07	180.65 ± 4.78	179.86 ± 5.13
350	178.89 ± 6.64	180.47 ± 5.21	180.32 ± 5.71

## Data Availability

The data presented in the current study are available in the article and in the associated Supplementary Material.

## Supplementary Materials

The following supporting information can be downloaded at: https://www.mdpi.com/article/10.3390/ijms23042138/s1.

## Abbreviations

The following abbreviations are used in this manuscript:

HB	hydrogen bond
IR	infrared spectroscopy
FTIR	Fourier transform infrared spectroscopy
NDE	nonlinear dielectric effect
MD	molecular dynamics
CPMD	Car–Parrinello molecular dynamics
PIMD	path integral molecular dynamics
CCDC	Cambridge Crystallographic Data Centre
RMSD	root mean square deviation
RDF	radial distribution function
PBC	periodic boundary conditions
DFT	density functional theory
pmf	potential of mean force
