# Supplementary material for: Inside out Approach to Rotator State in Hydrogen-Bonded System—Experimental and Theoretical Cross-Examination in n-Octanol
Source: Int J Mol Sci. 2022 Feb 15;23(4):2138. doi: 10.3390/ijms23042138 (PMC8877557; doi:10.3390/ijms23042138)
Supplement: Supplementary file 1 [file ijms-23-02138-s001.zip › ijms-1565098-supplementary.pdf]

SUPPLEMENTARY MATERIAL

Inside Out Approach to Rotator State in Hydrogen Bonded System – Experimental and Theoretical Cross-Examination in n-Octanol

Michał Pocheć[1]*, Katarzyna M. Krupka[1], Jarosław J. Panek[1], Kazimierz Orzechowski[1], Aneta Jezierska[1]*

[1]University of Wrocław, Faculty of Chemistry, ul. F. Joliot-Curie 14, 50-383 Wrocław, Poland

Correspondence should be addressed to: michal.pochec@chem.uni.wroc.pl, aneta.jezierska@chem.uni.wroc.pl; tel.: +48 71 3757 224; fax: +48 71 3282 348

Table of contents:

I. **Figure S1.** Experimental spectra of n-octanol in: -84 ^{o}C, -80 ^{o}C, -61 ^{o}C and -45 ^{o}C.

II. **Figure S2.** Experimental spectra of n-octanol in: -43 ^{o}C, -35 ^{o}C, -31 ^{o}C, -28 ^{o}C and -25 ^{o}C.

III. **Figure S3.** Experimental spectra of n-octanol in: -24 ^{o}C, -22 ^{o}C, -21 ^{o}C, -20 ^{o}C and -19 ^{o}C.

IV. **Figure S4.** Experimental spectra of n-octanol in: -18 ^{o}C, -17 ^{o}C, -16 ^{o}C, -15 ^{o}C and -14 ^{o}C.

V. **Figure S5.** Root mean square deviation (RMSD) of the non-hydrogen atoms along the classical MD trajectories. The experimental X-ray structure [1] was used as a reference. For each subsequent temperature, the RMSD graph was raised by 1, so that the graphs do not overlap; in fact, the RMSD for each simulation oscillates around 1 Å. The last graph for 417 K (green line) is scaled by the factor of 0.01 - the RMSD quickly grows to over 200 Å, indicating collapse of the crystal ordering and transition to the liquid phase.

VI. **Figure S6.** Temperature dependence of dipole moment power spectra corresponding to the IR absorptions - results of Car-Parrinello molecular dynamics simulations.

VII. **Figure S7.** Temperature dependence of vibrational signatures of the hydroxyl proton atoms - results of Car-Parrinello molecular dynamics simulations.

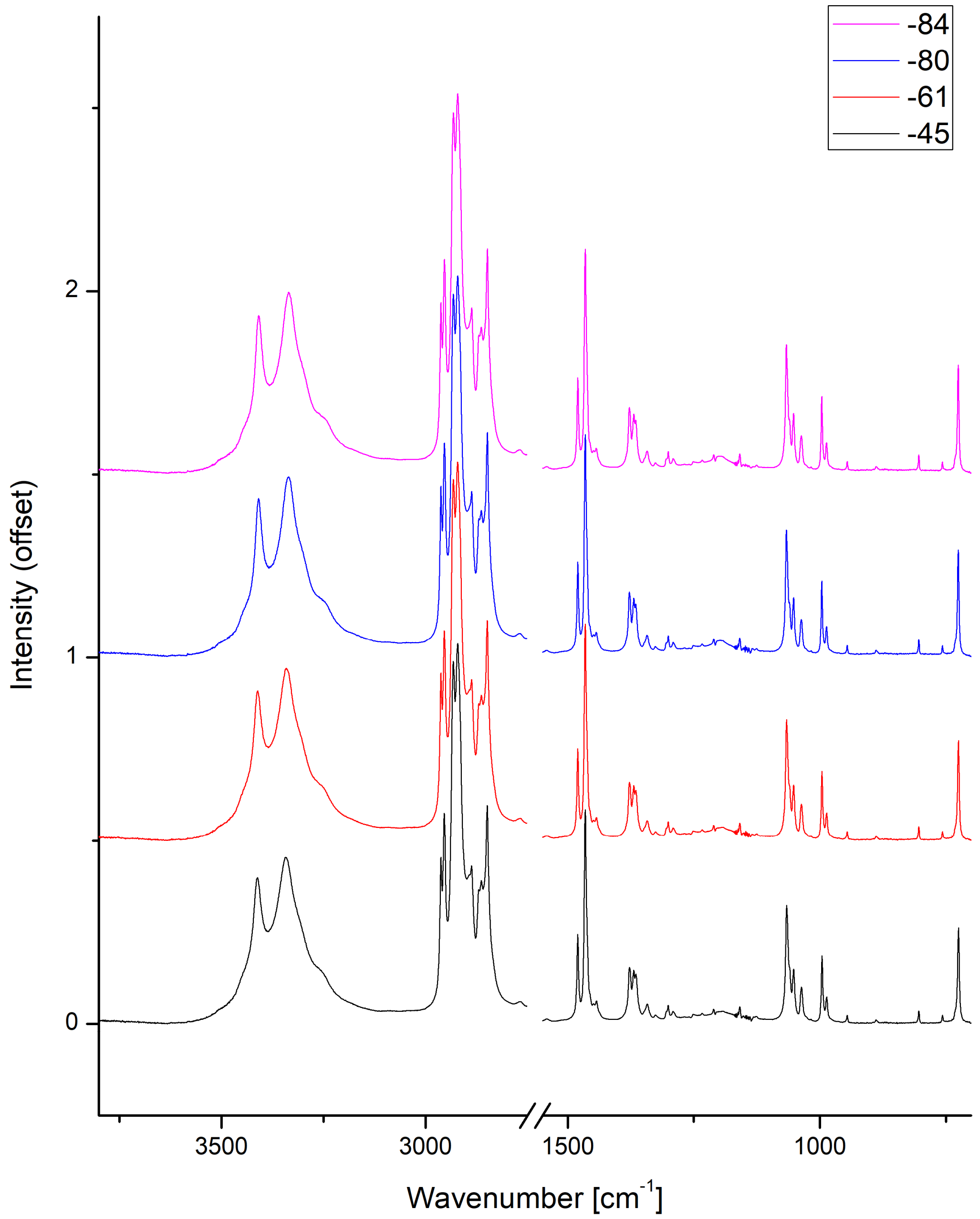

Figure S1. Experimental spectra of n-octanol in: -84 ^{o}C, -80 ^{o}C, -61 ^{o}C and -45 ^{o}C.

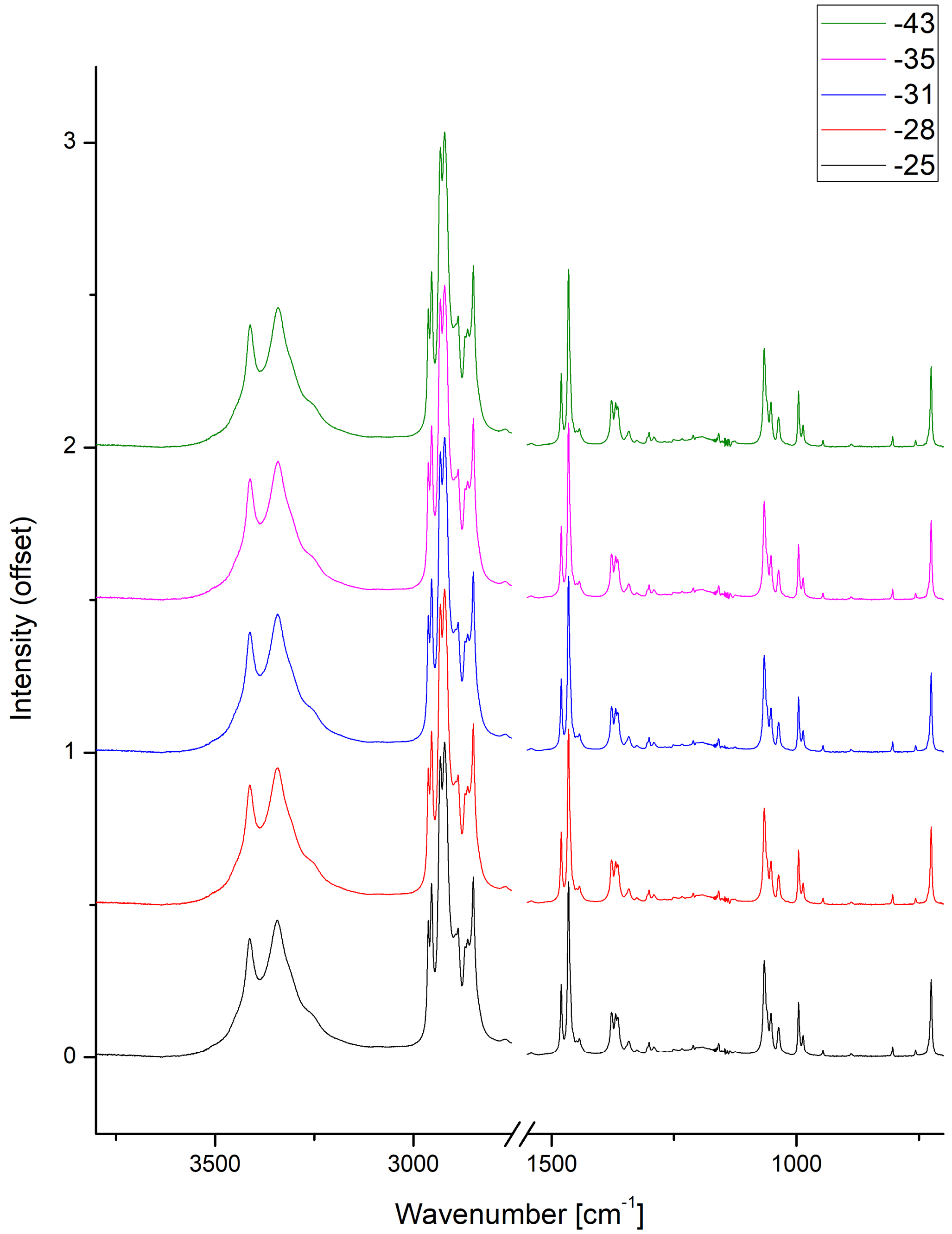

Figure S2. Experimental spectra of n-octanol in: -43 ^{o}C, -35 ^{o}C, -31 ^{o}C, -28 ^{o}C and -25 ^{o}C.

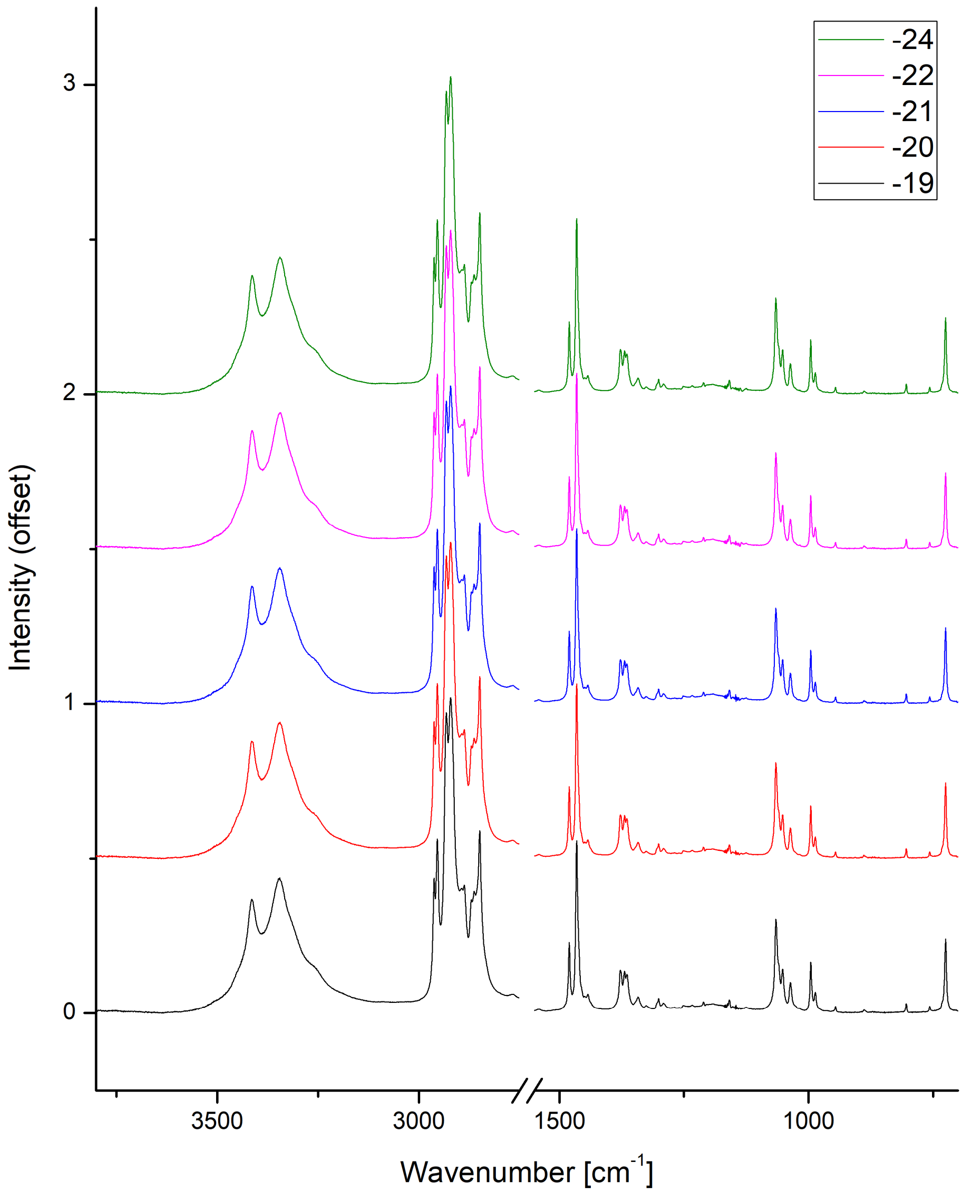

Figure S3. Experimental spectra of n-octanol in: -24 ^{o}C, -22 ^{o}C, -21 ^{o}C, -20 ^{o}C and -19 ^{o}C.

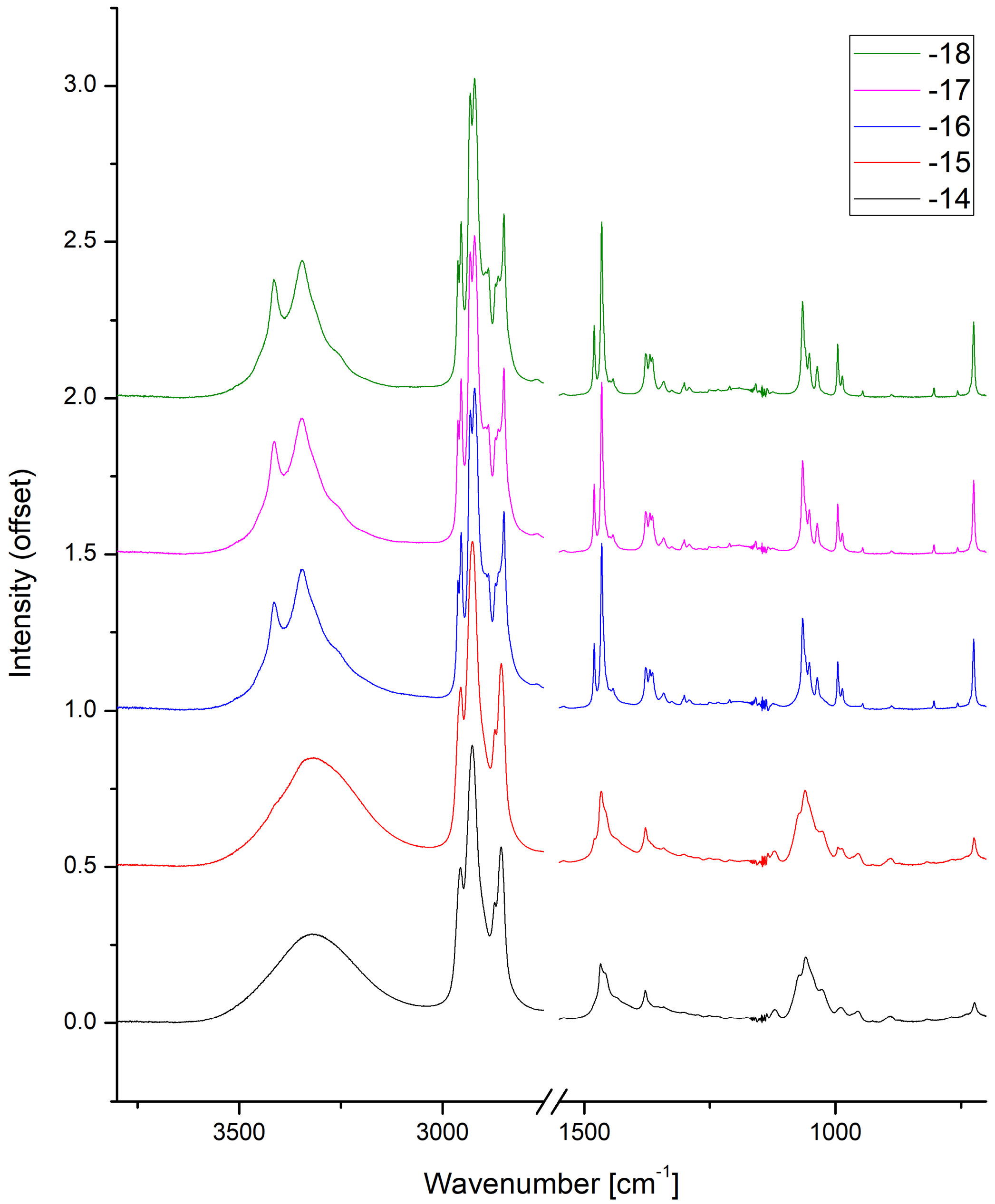

Figure S4. Experimental spectra of n-octanol in: -18 ^{o}C, -17 ^{o}C, -16 ^{o}C, -15 ^{o}C and -14 ^{o}C.

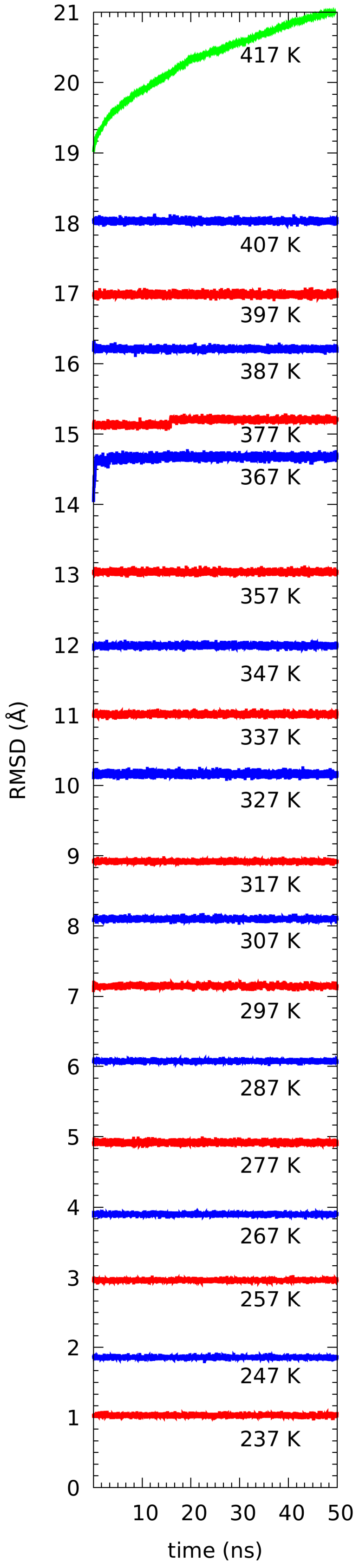

Figure S5. Root mean square deviation (RMSD) of the non-hydrogen atoms along the classical MD trajectories. The experimental X-ray structure [1] was used as a reference. For each subsequent temperature, the RMSD graph was raised by 1, so that the graphs do not overlap; in fact, the RMSD for each simulation oscillates around 1 Å. The last graph for 417 K (green line) is scaled by the factor of 0.01 - the RMSD quickly grows to over 200 Å, indicating collapse of the crystal ordering and transition to the liquid phase.

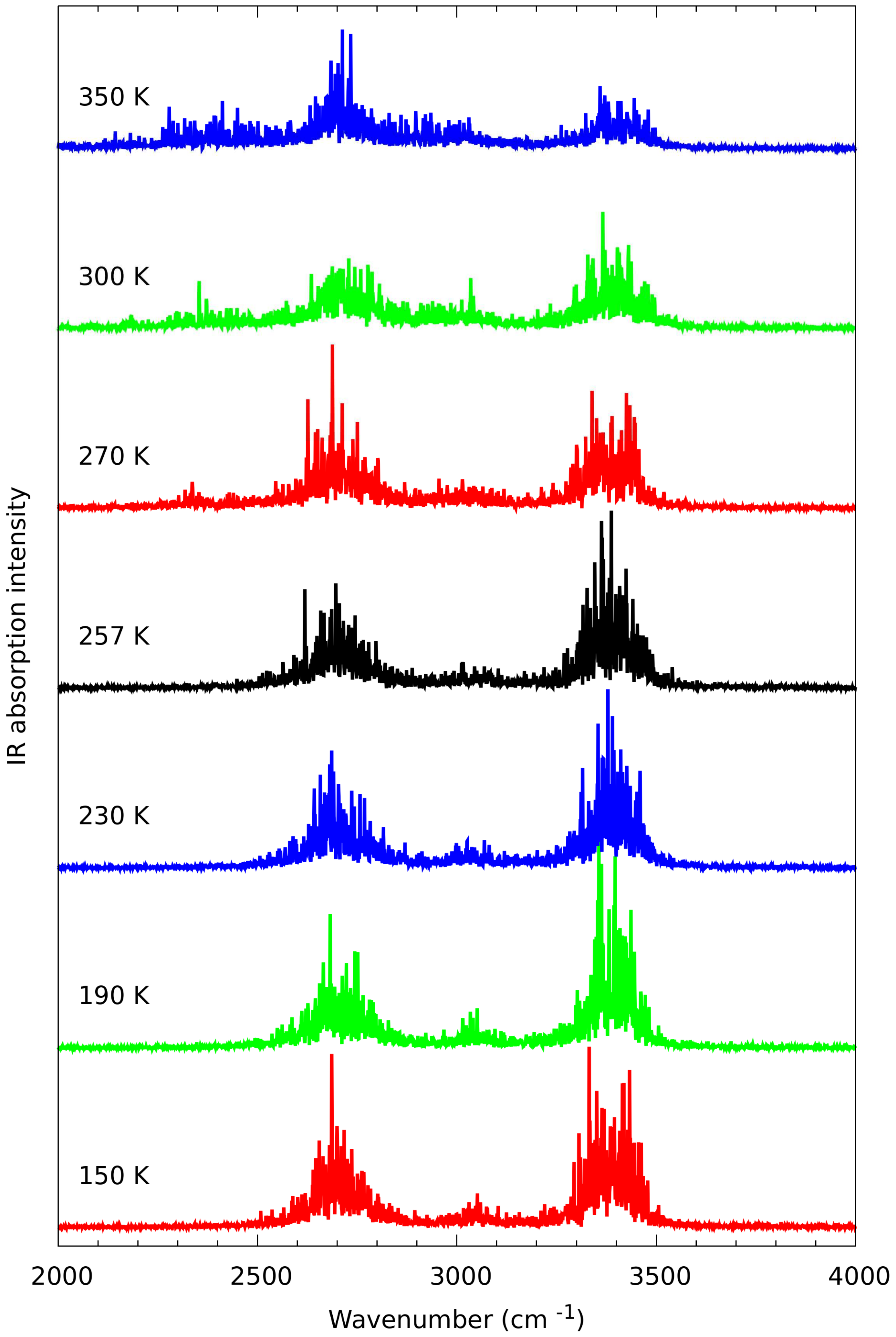

Figure S6. Temperature dependence of dipole moment power spectra corresponding to the IR absorptions - results of Car-Parrinello molecular dynamics simulations.

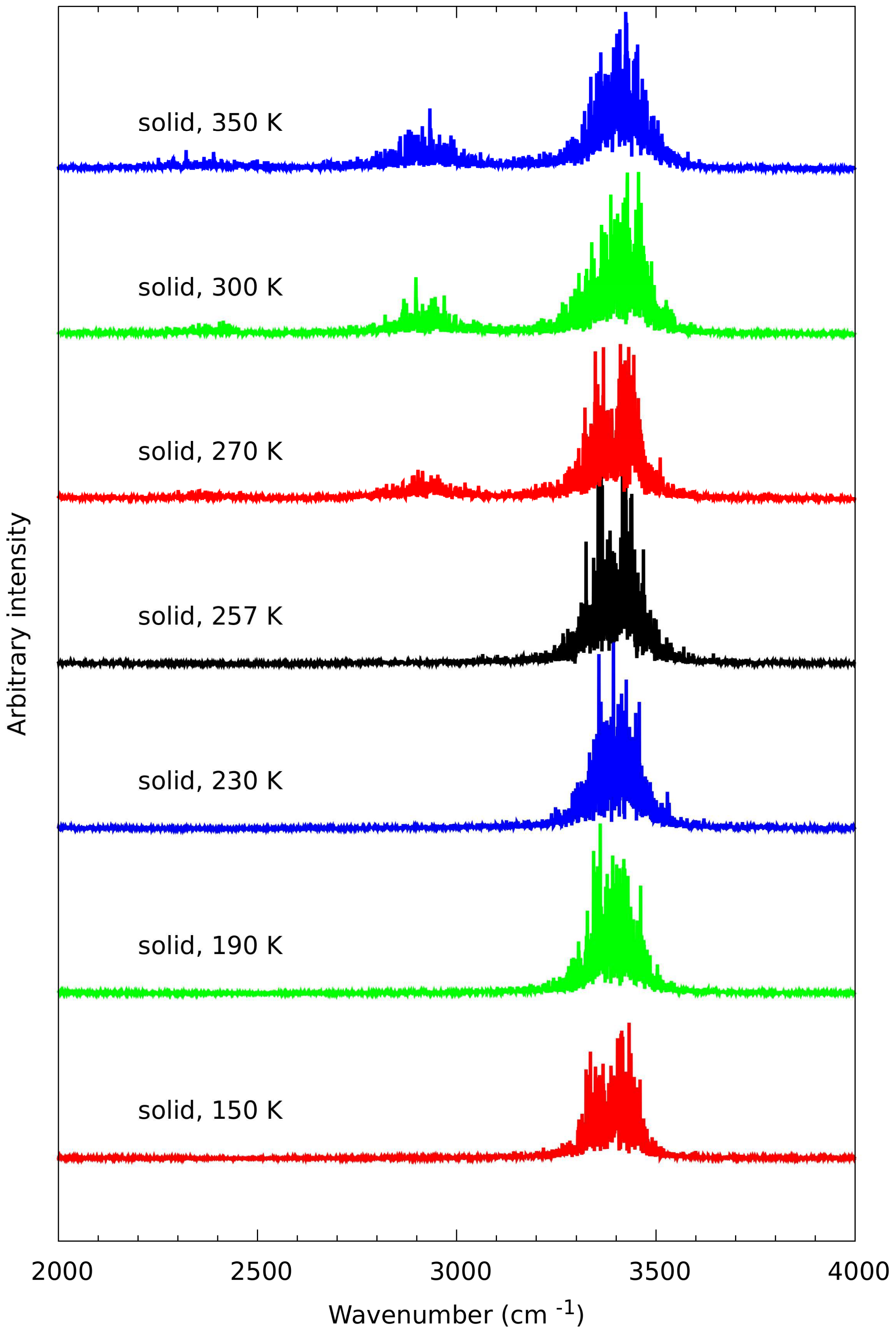

Figure 7. Temperature dependence of vibrational signatures of the hydroxyl proton atoms - results of Car-Parrinello molecular dynamics simulations.

References:

[1] Howard A. Shallard-Brown, David J. Watkin, Andrew R. Cowley, n-Octanol. *Acta Crystallogr. E*, **2005**, *61*, o213-o214.
